# Assessing Chat Generative Pretrained Transformer-4's clinical utility in acute compartment syndrome: a comprehensive evaluation of accuracy, completeness, and readability

**DOI:** 10.1590/1806-9282.20250892

**Published:** 2025-12-15

**Authors:** Öner Kılınç, İdris Demirtaş

**Affiliations:** 1 Ministry of Health Mersin City Education and Research Hospital, Department of Orthopedics and Traumatology – Mersin, Turkey.

**Keywords:** Compartment syndromes, Traumatology, Artificial intelligence, Large language models

## Abstract

**OBJECTIVE:**

Artificial intelligence tools like Chat Generative Pretrained Transformer-4 are increasingly used in clinical decision-making, but their reliability for acute compartment syndrome remains understudied. The aim of the study was to evaluate Chat Generative Pretrained Transformer-4's accuracy, completeness, and quality in responding to acute compartment syndrome-related queries, addressing gaps in artificial intelligence-assisted medical information.

**METHODS:**

Chat Generative Pretrained Transformer-4 was given 60 questions (40 open-ended and 20 binary) that were taken from the American Academy of Orthopaedic Surgeons 2019 acute compartment syndrome guidelines. Responses were evaluated independently by two orthopedic specialists using the Quality Criteria for Consumer Health Information instrument (information quality), Flesch-Kincaid Reading Ease Score (readability), and Likert scales (accuracy and completeness). Inter-rater reliability (Cohen's kappa) was a statistical analysis.

**RESULTS:**

Chat Generative Pretrained Transformer-4 demonstrated high accuracy (95% for both question types) and completeness (mean scores: 5.92±0.8 [accuracy], 2.9±0.5 [completeness]). DISCERN scores were "excellent" (69–72), though source reliability was limited. Readability was "very difficult" (Flesch-Kincaid Reading Ease Score: 22.19), potentially hindering patient comprehension.

**CONCLUSION:**

Although Chat Generative Pretrained Transformer-4 is excellent at providing precise, high-quality acute compartment syndrome information, its complicated language and lack of credible sources make it difficult for wider adoption. To improve clinical utility and patient education, readability and transparency must be given top priority in future artificial intelligence developments.

## INTRODUCTION

Acute compartment syndrome (ACS) is a common pathophysiological complication resulting from trauma or tissue ischemia^1^. ACS was selected as the test case in this study because it is a time-critical orthopedic emergency in which delays in diagnosis and treatment can lead to irreversible neuromuscular damage, limb loss, or even death. Its clinical management typically requires rapid recognition of signs and symptoms and prompt, decisive intervention^2^. Therefore, ACS is considered an ideal scenario for evaluating how accurately, comprehensively, and practically artificial intelligence (AI)-generated medical information can guide in high-risk situations. Diagnosing ACS is often challenging, and the only effective treatment is decompressive surgical fasciotomy^1^. The American Academy of Orthopaedic Surgeons (AAOS) published updated recommendations for the diagnosis and treatment of ACS in 2019^3^.

The goal of AI, a subfield of computer science, is to build machines that can autonomously collect data and carry out tasks using digital information^4^. A branch of AI called machine learning uses algorithms to forecast results and glean insights. Sophisticated language models such as OpenAI's Chat Generative Pretrained Transformer (ChatGPT), which has been extensively trained on a large text dataset, have been developed as a result of recent developments in AI and data-learning technologies^5,6^. An important milestone was reached in March 2023 with the announcement of GPT-4, which is based on ChatGPT's framework^7^.

AI is essential in disease diagnosis, treatment recommendations, and clinical decision-making, improving efficiency and reducing errors. In emergencies such as ACS, AI systems can rapidly evaluate patient conditions and facilitate accurate treatment decisions. To the best of our knowledge, this is the first study to evaluate ChatGPT-4's performance in providing clinical guidance for ACS based on established guidelines.

The purpose of this study was to evaluate ChatGPT-4's accuracy and comprehensiveness in providing information about ACS by the 2019 AAOS guidelines. This study aims to investigate the potential benefits and drawbacks of ChatGPT-4 as an additional tool for medical professionals managing ACS in emergencies by assessing AI-generated answers to clinically relevant questions.

## METHODS

### Study design

This study assesses ChatGPT-4's efficacy in treating ACS by the 2019 AAOS guidelines^3^. The study did not require informed consent or ethical approval because it did not use human or animal subjects. The Declaration of Helsinki's ethical guidelines were followed in every procedure, guaranteeing the study's moral integrity and adherence to global norms. In addition, the study was conducted by emerging AI-specific ethical principles, including transparency regarding the AI's role in content generation, acknowledgment of AI limitations, and avoidance of misleading or unverifiable claims.

### Formation and evaluation of queries

A set of 60 questions based on the AAOS 2019 guidelines for managing ACS was developed by orthopedic specialists ÖK and İD. They independently verified the datasets. ChatGPT's responses were evaluated by them and compared statistically. The guidelines were not uploaded to ChatGPT, and questions were not pre-tested on the platform. All questions were presented to ChatGPT-4 using standardized and fixed phrasing to minimize variability in responses due to differences in wording. No rephrasing, follow-up prompts, or iterative clarification were used, ensuring that each query was delivered in its original, predefined form.

To evaluate AI's accuracy in recognizing right answers and verifying claims regarding ACS management, the questions were divided into 40 open-ended (Group 1) and 20 binary (true/false) questions (Group 2). Diagnostic standards, therapeutic approaches, and follow-up procedures were among the subjects covered.

Questions were sent to ChatGPT-4 on April 16, 2025, and answers were graded on a Likert scale with 1 denoting "completely incorrect" and 6 denoting "completely correct." Using a three-tiered scoring system, completeness scores assessed whether AI responses contained the required information: 1 for "Inadequate," 2 for "Adequate," and 3 for "Comprehensive."

Completeness scores were used to assess how well AI responses addressed questions and whether they offered the information required about a research topic. Three levels of scoring were used: 1 for "Inadequate," which means that some aspects were covered but that important components were missing; 2 for "Adequate," which means that all aspects were covered with the bare minimum of information required; and 3 for "Comprehensive," which indicates that all aspects were addressed with additional pertinent information.

In open-ended situations, ChatGPT-4 sought to bolster its responses with justifications for Likert-scale evaluation scoring. A well-known instrument for evaluating the caliber of written patient information on medical management options is the Quality Criteria for Consumer Health Information (DISCERN) Scale. It consists of 16 questions divided into three categories: overall quality, treatment information, and reliability. The maximum score is 80; scores above 70 are considered "excellent," and scores above 50 are considered "good." Questions are scored on a scale of 1–5, with 1 denoting "definite no" and 5 denoting "definite yes.^8^"

Out of the 60 questions, 44 (73.3%) dealt with ACS diagnostics, 16 (26.7%) with treatment, and none were solely about follow-up or long-term monitoring. The 2019 AAOS guidelines, which place the greatest emphasis on diagnostic evaluation, were reflected in this distribution. Each question made an equal contribution to the accuracy, completeness, and DISCERN analyses; no additional statistical weighting was used.

After independently rating each response on the DISCERN scale, accuracy, and completeness, two authors (ÖK and İD) had direct conversations about any discrepancies until they agreed on the final scores.

The Flesch-Kincaid Reading Ease Score (FRES) was used to evaluate each group's responses for readability. The formula is used to determine the FRES. On a scale of 0–100, the outcomes are assessed. Scores fall into the following categories: 90–100: Very easy (grade 5 and below); 80–89: Easy (grade 6); 70–79: Average (grades 7–8); 60–69: Average (grade 9); 50–59: Difficult (grades 10–12); and 0–49: Very difficult (university level and above)^9^.

### Statistical analysis

Version 25.0 of Statistical Package for the Social Sciences for Windows was used to perform statistical analyses. The Shapiro-Wilk and Kolmogorov-Smirnov tests were used to determine whether the variables were normal. The data is considered to have a normal distribution if the p-value in the Kolmogorov-Smirnov test is greater than 0.05. Non-parametric tests, such as the Mann-Whitney U test, were used when the data did not follow a normal distribution. The means and standard deviations of the gathered data were among the descriptive statistical analyses that were carried out. Cohen's kappa statistic was used to assess inter-rater reliability. Statistical significance was defined as a p<0.05.

## RESULTS

Our study assessed 20 binary (true/false) questions in Group 2 and 40 open-ended questions in Group 1. Following analysis of the Group 1 questions, the accuracy scores were as follows: 38 (95%) questions received Likert 6 points, one (2.5%) question received Likert 5 points, and one (2.5%) question received Likert 4 points. 5.92±0.8 was the average accuracy score. Completeness ratings: four (10%) questions were given Likert 2 points, while 36 (90%) questions were given Likert 3 points. 2.9±0.5 is the mean completeness score.

Nineteen (95%) of the Group 2 questions had Likert 6 points, and one (5%) had Likert 5 points, according to the accuracy scores. For completeness, 19 (95%) questions received Likert 3 points, while one (5%) question received Likert 2 points. The average accuracy score was 5.95±0.2. 2.95±0.2 is the mean completeness score. Table 1 shows how the groups’ Likert scale scores for completeness and accuracy were distributed.

**Table 1:** Likert scale score distribution for accuracy and completeness between groups.

	Group 1 (n=40)		Group 2 (n=20)	
	Accuracy, n (%)	Completeness, n (%)	Accuracy, n (%)	Completeness, n (%)
Likert-1	0		0	0
Likert-2	0	4 (10)	0	1 (5)
Likert-3	0	36 (90)	0	19 (95)
Likert-4	1 (2.5)		0	
Likert-5	1 (2.5)		1 (5)	
Likert-6	38 (95)		19 (95)	

Group 1: Open-ended questions, Group 2: Binary (true/false) questions.

Three primary subcategories were used to analyze DISCERN scores in detail: overall assessment (question 16), quality of treatment (questions 9–15), and reliability (questions 1–8). A total DISCERN score of 66 was obtained for open-ended questions, with reliability scoring 30/40, treatment quality scoring 32/35, and overall assessment scoring 4/5. Reliability, treatment quality, and overall assessment scores for binary (true/false) questions were 33/40, 34/35, and 5/5, respectively, for a total score of 72. These findings show that ChatGPT-4 consistently and highly performed across all DISCERN criteria. Because ChatGPT-4's responses lacked cited sources, some specific items—especially those assessing the reliability of sources or the presence of references—received comparatively lower scores than the overall "excellent" DISCERN scores.

There was no difference between groups 1 and 2 when assessed based on DISCERN scores, accuracy, and completeness (p=0.864, p=0.942, and p=0.680, respectively) (Table 2).

**Table 2:** Evaluation of accuracy, completeness, and DISCERN scores between groups.

	Group 1 (n=40)	Group 2 (n=20)	p-value
Accuracy, mean±SD	5.92±0.8	5.95±0.2	0.864
Completeness, mean±SD	2.9±0.5	2.95±0.2	0.942
DISCERN	69	72	0.680

DISCERN: Quality Criteria for Consumer Health Information, Group 1: Open-ended questions, Group 2: Binary (true/false) questions, DISCERN scores are presented as total scores (maximum possible score=80). SD: standard deviation.

In accordance with McHugh's classification, the inter-rater reliability as measured by Cohen's kappa coefficient was 0.94 for accuracy, 0.96 for completeness, and 0.98 for DISCERN, indicating excellent agreement (p<0.001 for all).

According to the FRES classification, the reading ease score of 22.19 was deemed "very difficult: university level."

## DISCUSSION

In our study, ChatGPT-4 responded to 40 open-ended and 20 binary (true/false) questions based on the 2019 AAOS guidelines for ACS. These responses were evaluated by two orthopedic specialists in terms of accuracy, completeness, and quality using the DISCERN instrument. ChatGPT-4 achieved excellent scores for reliability, accuracy, and completeness in both question groups. The only drawback was the identification of insufficient references according to the DISCERN scale. Additionally, the readability score, categorized as "university level" on the FRES, suggests that the content may not be easily accessible to a broader audience. To the best of our knowledge, this is the first study in the literature to evaluate the use of AI in ACS.

The literature shows that AI has been studied in many areas of orthopedic practice. One such study is that of Megalla et al., which evaluated ChatGPT and Google in relation to rotator cuff repair. Our study differs from Megalla et al.'s in three main ways: we examined ACS, a time-critical emergency, rather than an elective procedure; we used 60 structured, guideline-based questions instead of popular search queries; and we applied quantitative metrics such as accuracy, completeness, DISCERN, and FRES, whereas they used qualitative rating categories. Despite these methodological differences, both studies found that ChatGPT delivered mostly accurate and clinically appropriate responses, highlighting its potential as a medical information tool. However, our results also underscore the challenges of reference completeness and readability in high-complexity topics, whereas Megalla et al. emphasized ChatGPT's advantage over Google in providing more academically sourced content^10^.

ChatGPT has been evaluated in many areas of orthopedics, including rotator cuff repair, anterior cruciate ligament reconstruction, total hip arthroplasty, and spinal surgery. The results show that ChatGPT's responses to common patient questions were mostly rated as "excellent or satisfactory" and provided clear, evidence-based answers^11–13^. However, beyond orthopedic conditions, ChatGPT-4 has also been shown to provide satisfactory information for diagnosis and treatment in many other health areas, such as rheumatoid arthritis, the use of anti-rheumatic drugs during pregnancy, prostate cancer and benign prostatic hyperplasia, cardiac arrest, and cardiopulmonary resuscitation^14–17^. Our study confirms that ChatGPT-4 provides excellent information for ACS, consistent with high accuracy, completeness, and DISCERN scores aligned with the literature. While no statistically significant difference was found between open-ended and dichotomous questions in DISCERN scores, the richness of content was more pronounced in open-ended questions. This finding suggests that open-ended questions can better reveal the quality of information provided by AI models.

When examining the subheadings that contributed most to the high scores obtained in our study, we found that the headings evaluating information quality and clarity of treatment options made the greatest contribution. However, items related to source citations and information currency received relatively lower scores. This is related to ChatGPT-4's occasional failure to cite sources and provide incomplete references^18,19^. This phenomenon, known as "hallucination," has also been observed in orthopedic and health-related research^20^. To lessen the possibility of such "hallucinations," future AI systems should include real-time citation tools that can directly connect responses to authoritative guidelines or peer-reviewed sources. Integrating fact-checking algorithms and permitting source origin transparency could further increase reliability. Additionally, allowing users to verify information with reference lists or embedded hyperlinks at the time of response generation would boost confidence and encourage evidence-based decision-making.

Another important concern is the "reading level." Since the average reading level of adults in the United States does not exceed eighth grade, the readability of medical information generated by AI is very important. Institutions such as the National Institutes of Health (NIH) and Centers for Disease Control and Prevention (CDC) recommend that health materials be written at a sixth-grade level or below^21^. In our study, the FRES reading level was classified as "very difficult: university level." While this may not pose a problem for healthcare professionals, it could present a significant barrier in terms of patient education and general public information. Low FRES scores can negatively affect information transfer by reducing reader interest and comprehension^22–24^. For AI-based information sources to be effective patient communication tools, content must be simplified to suit the literacy level of the target audience. Therefore, future studies recommend evaluating AI content not only for medical accuracy but also for suitability for different levels of health literacy. To get around this limitation, future AI tools may incorporate adaptive language settings or simplified language tiers, which would enable content to be automatically adjusted to the reader's health literacy level. For example, different output modes could be offered to patients and healthcare professionals, with the latter emphasizing the use of short sentences, straightforward language, and the avoidance of technical medical jargon. Incorporating readability algorithms into the generation process could also guarantee that patient-facing outputs meet the sixth-grade reading levels recommended by the NIH and CDC.

This study has several limitations. First, despite being meticulously curated from the 2019 AAOS guidelines, the relatively small sample size of 60 questions might not fully represent the range of clinical scenarios seen in ACS. The findings’ generalizability may be impacted by this limitation, especially in cases of uncommon complications or atypical presentations. A more thorough assessment of ChatGPT-4's performance would be possible in future research using a larger and more varied set of questions, such as scenario-based and case-specific items. Second, there is a chance of subjective bias because the evaluation was based on the opinions of two orthopedic specialists. Even though there was a high degree of inter-rater agreement, future analyses could be made even more objective and robust by using blinded assessment protocols or adding more evaluators. Third, although generally regarded, the 2019 AAOS guidelines, upon which the questions were based, might not accurately represent the most recent data or changing clinical recommendations. In order to guarantee that AI performance is assessed in relation to the most recent standards of care, future research should take into account utilizing the most recent guidelines or combining several evidence-based sources. Additionally, changes in prompt phrasing or updates to the underlying model may cause ChatGPT-4's outputs to change over time. Because AI models are dynamic, results may not be entirely reproducible in subsequent evaluations, even though a standardized question structure was employed in this study to reduce such variability.

## CONCLUSION

According to the 2019 AAOS ACS guidelines, our research demonstrates that ChatGPT-4 is capable of providing extremely precise and comprehensive responses to both binary and open-ended questions. Excellent inter-rater reliability and high accuracy and completeness scores support AI's potential to provide pertinent medical information. Readability and source reliability are still issues, though. Although ChatGPT-4's responses frequently correspond with recent medical research, the absence of credible sources calls into question the accuracy of the data, especially when it comes to "hallucination." The text was classified as "very difficult: university level" by the reading ease score, suggesting a potential barrier to accessibility for non-specialists. Healthcare workers might not be hampered by this complexity, but patient education is made more difficult. Enhancing readability and expanding information accessibility for a range of audiences should be the main goals of future developments. Finally, the number of evaluated questions and the subjective nature of evaluations by a small group of specialists limit the insights of this study, underscoring the need for larger studies to validate these results and evaluate the usefulness of ChatGPT-4 in clinical settings. To fully comprehend the long-term effectiveness and safety of AI-generated medical information in practical settings, more research is necessary.

## Data Availability

The datasets generated and/or analyzed during the current study are available from the corresponding author upon reasonable request.
